# A Systematic Review of AI Performance in Lung Cancer Detection on CT Thorax

**DOI:** 10.3390/healthcare13131510

**Published:** 2025-06-24

**Authors:** Hao Min Cheo, Chern Yue Glen Ong, Yonghan Ting

**Affiliations:** 1 National University Hospital, Singapore 119074, Singapore; cheohaomin@gmail.com; 2 Department of Diagnostic Radiology, Tan Tock Seng Hospital, Singapore 308433, Singapore; glen_ong@ttsh.com.sg; 3 Department of Diagnostic Radiology, National University Hospital, Singapore 119074, Singapore

**Keywords:** artificial intelligence, computed tomography scan, deep learning, lung nodules, nodule detection

## Abstract

**Background:** The introduction of lung cancer screening (LCS) programmes will lead to a surge in imaging volumes and place greater demands on radiologists to provide timely and accurate interpretation. This increased workload risks overburdening a limited radiologist workforce, delaying diagnosis, and worsening burnout. Advancements in artificial intelligence (AI) models offer the potential to detect and classify pulmonary nodules without a loss in diagnostic performance. **Methods:** A systematic review of AI performance in lung cancer detection on computed tomography (CT) scans was conducted. Multiple databases like Medline, Embase, PubMed, and Cochrane were searched within a 12-year range from 1 January 2010 to 21 December 2022. **Results:** Fourteen studies were selected for this systematic review, with seven in the detection subgroup and eight in the classification subgroup. Compared to radiologists’ performance in the respective articles, the AI models demonstrated a higher sensitivity (86.0–98.1% against 68–76%) but lower specificity (77.5–87% against 87–91.7%) for the detection of lung nodules. In classifying the malignancy of lung nodules, AI models generally showed a greater sensitivity (60.58–93.3% against 76.27–88.3%), specificity (64–95.93% against 61.67–84%), and accuracy (64.96–92.46% against 73.31–85.57%) over radiologists. **Conclusion:** AI models for the detection and classification of pulmonary lesions on CT have the potential to augment CT thorax interpretation while maintaining diagnostic accuracy and could potentially be harnessed to overcome challenges in the implementation of lung cancer screening programmes.

## 1. Introduction

Lung cancer is a commonly diagnosed cancer and the leading cause of cancer death, responsible for almost 2.5 million new cases worldwide [1]. Furthermore, the World Health Organization (WHO) has estimated 1.8 million lung cancer deaths annually [2]. The overall 5-year survival rate of lung cancer is 17% and ranges from 70% for stage I to less than 5% for stage IV [3]. Across the world, lung cancer is amongst the top three diagnosed cancers in both men and women [4]. Stratified by sex, research suggests that women with lung cancer have a significant survival advantage over men, regardless of stages [5].

The American Cancer Society (ACS) recommends LCS with low-dose computed tomography (LDCT) for individuals aged 50 to 80 years who are current smokers or have a 20 or greater pack-year smoking history [6]. Globally, LCS guidelines align with those of the ACS due to the elevated risk of lung cancer in these populations, although there are slight variations in the recommended age ranges and smoking history criteria across different countries [7,8,9]. However, with LCS programmes being limited and carried out in an opportunistic manner [10], a robust and more extensive LCS programme is important because early-stage lung cancer is easier to treat and confers a more favourable 5-year survival rate [10,11].

There are three primary methods for LCS: chest X-ray (CXR), sputum cytology, and LDCT. Amongst these three, research has consistently shown that LDCT is the most effective test, and the use of LDCT for LCS effectively decreases the risk of death from lung cancer in current and former heavy smokers [12]. As such, LDCT has been established as the gold standard in the early detection of lung cancer [13].

However, the detection and characterisation of pulmonary nodules are laborious tasks, particularly when trying to detect subcentimetre lesions in an image cluttered with vessels and airways and when under time pressure [14]. If LDCT is adopted as a screening tool, with an accompanying increase in scan volumes, AI augmentation may be one of the avenues that could help to address the added workload [15]. Hence, augmentation with AI algorithms to aid in reading scans could potentially increase the efficiency of LCS and ensure that cancers are detected promptly and accurately [16].

Most lung cancer patients are diagnosed at an advanced stage [17], resulting in poor prognosis. In the United States, the implementation of LDCT screening programmes among smokers increased the diagnosis of stage I lung cancer while reducing that of stage IV. At the same time, the overall incidence of lung cancer remained steady, and the overall survival rates for lung cancer improved [18]. Similarly, studies on LCS programmes in Asian populations have found that including never-smokers in these programmes revealed that, numerically, there are more stage I lung cancer diagnoses among never-smokers than among ever-smokers [19].

Conventionally, CXR has been the modality of choice for LCS; however, it is not sufficiently sensitive in detecting lung cancer in its early stages and has not been shown to reduce lung cancer mortality [20,21]. Therefore, due to its increased sensitivity in detecting smaller lesions as opposed to CXR, LDCT has been deemed the optimal choice for LCS [22,23]. However, LDCT suffers from a high false positive rate of up to 49.3% for baseline screening [24]. This is because it may detect other non-malignant lesions, such as benign intrapulmonary lymph nodes or noncalcified granulomas [25]. These false positive results result in additional resources spent on further scans and invasive procedures, potentially increasing the economic burden. As such, the classification of lesions found through imaging is an equally crucial component of image interpretation, whether by a human reader or AI model.

Having a reliable prediction and classification model would streamline this entire process, though the role of AI in this context remains a subject of debate [26]. Currently in radiology, AI applications are being developed to optimise scanner time, reduce patient waiting times, and triage patients [27]. With the widespread usage of AI in the field of radiology, further research is being carried out to develop AI algorithms with substantial competencies to detect and classify nodules [28]. More specifically, AI models were used in LCS trials and accurately distinguished benign nodules from malignant ones, which in turn helped to reduce the delay between lung nodule detection and its definitive classification [29]. With the evident benefits of AI in diagnostic imaging, this systematic review hence focuses on analysing and assessing the diagnostic performance of existing AI models in the detection and classification of lung cancer through CT scans.

## 2. Methods

This systematic review was performed with reference to the Cochrane Handbook for Systematic Reviews [30] and the Preferred Reporting Items for Systematic Reviews and Meta-Analyses (PRISMA) statement guidelines [31].

### 2.1. Study Screening

Study selection was conducted independently by two reviewers, each with over five years of experience in radiology research. Articles were first screened by title and abstract, followed by full-text review. Exclusions were applied in accordance with the predefined criteria: studies not published in English, those that did not evaluate AI-based detection or classification of lung cancer through chest CT, studies that relied solely on open-source datasets without local or independent test cohorts, and publication types such as case reports, abstracts, guidelines, or consensus statements.

### 2.2. Search Strategy

An extensive search of the literature was conducted across six major databases: MEDLINE (Ovid), Embase (Ovid), PubMed, CINAHL, Cochrane Library, and Scopus. The search covered over a 12-year period from 1 January 2010 to 21 December 2022 and was limited to articles published in English.

We employed a combination of controlled vocabulary (e.g., MeSH terms) and free-text keywords, structured using Boolean operators to comprehensively identify relevant studies involving artificial intelligence (AI), lung cancer, and computed tomography (CT). The terms were applied to the title, abstract, and subject heading fields. The search strings were adapted for syntax differences across databases.

The search string used in PubMed is as follows:

(“Lung Neoplasms”[MeSH] OR “lung cancer” OR “pulmonary neoplasm*” OR “lung nodule*”)

AND

(“Tomography, X-Ray Computed”[MeSH] OR “computed tomography” OR “CT scan” OR “CAT scan”)

AND

(“Artificial Intelligence”[MeSH] OR “deep learning” OR “machine learning” OR “computer vision” OR “neural network*”)

This strategy ensured the comprehensive retrieval of studies evaluating the AI-based detection or classification of lung cancer using chest CT. The full list of search strings for each database is available upon request.

### 2.3. Eligbility

**Figure 1:**
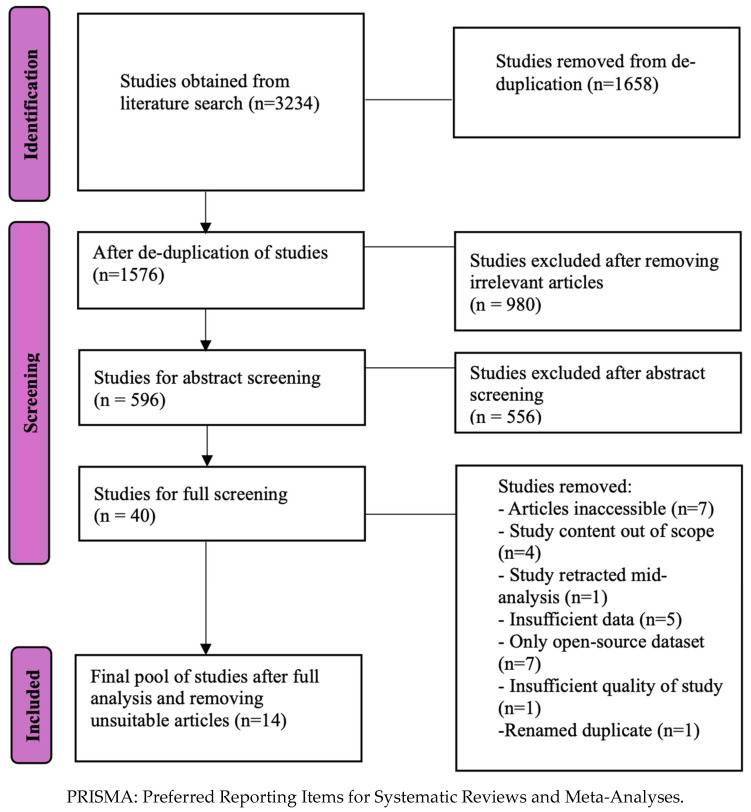
PRISMA flow chart demonstrating the literature review process.

In the initial search for studies, we obtained 3234 articles, of which 1658 were duplicates and removed. From the remaining 1576 articles, 980 of them were deemed irrelevant and removed as well. The reviewers then first screened the remaining 596 articles for potential inclusion by their titles and abstracts and excluded 556 articles. The remaining 40 articles were then reviewed in their entirety to confirm their inclusion (refer to Figure 1).

### 2.4. Data Extraction

After selecting the relevant studies, the reviewers separately identified the types of data required to facilitate an effective evaluation of each AI model’s performance. When studies included human readers, their performance was compared with that of the respective AI models. The values and information extracted from the studies were as follows:

- Title of the article;
- Names of authors;
- Name of AI model used;
- Year published;
- AI sensitivity, specificity, accuracy, and area under curve (AUC), together with human reader (radiologists) values of these categories, where provided;
- Number of patients and nodules;
- Focus on detection or classification.

The information extracted was then arranged into a table (Table 1). The sensitivity, specificity, accuracy, and AUC allowed for an assessment of the AI algorithm in terms of its lung nodule CT detection ability, whereas the numbers of patients and nodules were mostly collected as a reference but also to generate sensitivity, specificity, and accuracy values if they were not initially provided. Lastly, defining the type of study as either detection- or classification-focused was necessary to place them into one of two similarly named subgroups. It was necessary to split the studies into these two subgroups due to them exploring different stages of nodule analysis, along with different deep machine learning techniques, to achieve their objective.

### 2.5. Data Analysis

Data analysis was conducted by evaluating the range, mean, and standard deviation of values across each individual metric for both the detection and classification sub-groups. A meta-analysis was not conducted as this study was diverse in terms of study designs, population groups, and outcome measures.

## 3. Results

### 3.1. Baseline Characteristics

A total of 10,217 nodules across 14 studies were analysed [32,33,34,35,36,37,38,39,40,41,42,43,44,45]. Seven studies were focused on the detection of pulmonary nodules [32,33,34,35,36,37,38] and eight studies were focused on the classification of pulmonary nodules [37,39,40,41,42,43,44,45], with one study presenting with data on both detection and classification [37].

### 3.2. Detection of Lung Nodules

The seven studies for detection displayed varying amounts of data completion for the selected metrics in this research [32,33,34,35,36,37,38]. While all studies reported sensitivity values for the AI models they used, only some provided specificity and accuracy values for the AI models, as well as the radiologists’ sensitivity and specificity. Additionally, there was a noticeable lack of data for other metrics, with only Hsu et al., 2021 [36], providing a radiologist AUC value and none reporting values for AI AUC or radiologist accuracy. Also, it is worth noting that not all studies provided a 95% confidence interval for their data.

Overall, all implemented AI models demonstrated high sensitivity values. In the studies that included AI sensitivity values, the AI algorithms outperformed radiologists, which suggests that AI has the potential to accurately identify lung nodules in CT thorax.

However, most AI models generally performed poorer than radiologists in terms of specificity. The AI models had values ranging from 77.5% to 87%, which either matched those of radiologists, as seen in a study by Hsu et al., 2021 [36], or fell short, as in a study by Kozuka et al., 2020 [38]. This is also evident in a study by Cui et al., 2022 [35], where despite not providing a specificity value, the study reported an unusually high false positive rate of 359 false positives amongst a population totalling 262 nodules.

On the contrary, though a few studies provided high accuracy values for their AI models of between 85.71% to 99.02%, none of the studies provided the radiologists’ accuracy values for comparison (Table 2 and Table 3).

### 3.3. Classification of Lung Nodules

The results for the eight studies in the classification subgroup were as heterogeneous as the detection subgroup [36,38,39,40,41,42,43,44] (Table 4 and Table 5). Some measurements showed almost no values obtained, while radiologist AUC displayed no values at all.

Though both the AI models and radiologists had high sensitivity values, the values displayed by the AI models had a greater range, from 60.58% to 93.3%, compared to radiologists’ sensitivity values, ranging from 76.27% to 86.7%.

Similarly, for specificity values, AI models displayed a wider range of 64% to 95.93%, while radiologists displayed a smaller range of 61.67% to 84%. Despite the wider range, AI models generally classified the different lung nodules with greater specificity.

The accuracy for AI was mentioned in five studies, with values ranging from 64.96% to 92.46%. Though the accuracy for radiologists was only mentioned in two studies, with values ranging from 73.31% to 85.57%, the accuracy value for radiologists was higher than the accuracy for AI at 73.31% over 64.96% for the AI model DenseNet used by Qiu et al., 2022 [39].

## 4. Discussion

This systematic review demonstrates that AI models can achieve strong diagnostic accuracy in detecting lung cancer through chest CT scans. As a non-invasive approach, such AI models can support radiologists by aiding in the early detection and classification of lung cancer, which are crucial for timely diagnosis, effective treatment, and improved survival rates.

### 4.1. AI Performance

The National Lung Screening Trial (NLST) demonstrated that LDCT screening for lung cancer reduced mortality by 20% [25]. The accurate interpretation and detection of lung cancer in these scans remain challenging due to the three-dimensional complexity of CT images and the breadth of information contained within, which contributes to missed suspicious nodules [46]. Small nodules less than one centimetre in diameter may be especially difficult to distinguish from ordinary anatomic structures within the lungs [47]. Nonetheless, research has been carried out to compare the accuracy of nodule detection between experienced radiologists and AI algorithms. The findings reveal that AI algorithms play a valuable role in identifying nodules that even experienced radiologists may overlook, highlighting their potential in enhancing diagnostic accuracy [48]. Therefore, with the occurrence of errors from radiologists’ readings, there is a greater need for AI models to be employed, be it as a concurrent reader or a second reader [49].

It is also worth noting that most of the studies involved were not LDCTs. This would likely change the AI performance as the technical factors of the scans are different.

The detection subgroup in this systematic review highlighted high lung nodule detection rates, with impressive sensitivity that consistently outperformed radiologists across all applicable studies. This indicates that there are significant advantages in using AI to detect lesions on lung CTs, such as the ability to handle increasing case volumes with detection performance comparable to an experienced radiologist. Nonetheless, the review also revealed the need for further machine learning development, as studies showed a lower average specificity for AI models. As radiologists outperformed the AI models in this metric, the lower specificity rates of AI models could potentially lead to a high number of false positives, as seen in a study by Cui et al., 2022 [35]. If implemented clinically, such AI models can lead to unnecessary testing and increased workload for healthcare systems.

In the classification subgroup in this review, results comparing AI models with human readers in terms of specificity, sensitivity, and accuracy were mixed. Unlike the detection subgroup, where AI demonstrated clear superiority in sensitivity, AI in lung nodule classification still requires further development, as demonstrated by the slightly lower average result. Though lower in sensitivity, AI models managed to achieve better classification capabilities in the specificity and accuracy metrics. Nonetheless, the continuous improvement of classification capabilities is essential as it represents the next step after detection in automated reading. Having AI algorithms that can accurately distinguish between benign and malignant tumours, for example, can save significant resources and time that will otherwise go toward further testing if a tumour is detected in a patient.

### 4.2. Determinants of Health on Lung Cancer

Socioeconomic Status (SES) is a well-documented determinant of health that significantly influences lung cancer incidence and outcomes. Individuals residing in lower SES communities often face a disproportionate burden of disease, including higher rates of cancer [50]. These disparities are closely linked to structural inequalities, such as limited access to quality healthcare, preventative services, and early detection programmes, particularly in rural and underserved urban areas [51,52].

The financial strain associated with basic living expenses in these populations can discourage individuals from seeking medical care, especially when health concerns compete with immediate economic needs. Additionally, lower SES groups are less likely to take medical leave, often working through illness due to job insecurity or a lack of paid sick leave [53], potentially delaying diagnosis and treatment. Moreover, individuals from disadvantaged backgrounds may face barriers to accessing LDCT screening even if AI tools reduce radiology bottlenecks.

Environmental and occupational exposures are also contributors to increased lung cancer risk, particularly among populations with low SES who tend to reside near hazardous industrial sites like waste disposal facilities, power plants, and superfund locations, which are associated with chronic exposure to carcinogens [54]. In occupational settings, individuals in lower-paying, manual labour-intensive jobs—such as construction, mining, painting, and chimney sweeping—are frequently exposed to substances like asbestos, diesel exhaust, silica, and coal tar, which have been implicated in increased lung cancer risk [55,56,57]. Importantly, these occupational exposures elevate lung cancer risk in both smokers and non-smokers, though the risk is significantly amplified in individuals who smoke [57].

These findings show the cumulative impact of social and environmental factors in heightening lung cancer risk, especially in disadvantaged populations, where people tend to have reduced access to timely medical care and healthcare resources and occupy high-risk jobs while facing greater exposure to hazardous substances.

### 4.3. Difference in Methods of Studies

The primary issue in analysing these studies was the heterogeneity of data provided. As observed in both subgroups, data for certain metrics was insufficiently populated, as each study focused on different aspects of AI algorithm performance. Additionally, not all studies compared their AI models to radiologists’ readings, or they offered incomplete data, making it difficult to fully assess the models’ capabilities without a consistent baseline for comparison.

Also, the methodologies’ and studies’ focus varied widely, which may indirectly impact data congruence across the studies. For instance, some studies in the detection subgroup tested the AI model only on CT images with nodules, while others included control images without nodules, affecting the ability to use data like specificity. Additionally, while some studies measured performance per CT scan, others measured it per nodule, meaning multiple nodules can appear in one scan, complicating the comparison of results. In the classification subgroup, the range of classifications, benign and malignant or degrees of malignancy, introduced further challenges, as these may represent different stages of progression in nodule analysis.

### 4.4. Limitations of AI Models

Across the included studies, AI models exhibited substantial architectural variability. Convolutional Neural Networks (CNNs) such as ResNet, DenseNet, and Faster R-CNN were frequently used for detection tasks due to their proficiency in spatial feature extraction from volumetric CT data.

Classification models often employed ensemble methods (e.g., DenseNet + AdaBoost) or hybrid 2D/3D CNNs to account for the morphological complexity of nodules. However, few studies reported detailed preprocessing workflows (e.g., normalisation, segmentation), which are known to significantly affect performance. The lack of standardised evaluation protocols and the variable use of radiologist comparators further complicate benchmarking efforts across models.

One other significant limitation of AI models for detecting lesions on CT scans is their lack of transparency in decision-making, making it challenging for radiologists to validate their findings [58]. This lack of insight can pose issues when the AI model identifies lesions that radiologists may overlook, particularly in life-or-death scenarios. This limitation may be solvable through the development of explainable AI (XAI).

XAI methods may potentially address this challenge, through providing insights into the AI’s decision-making process, as well as enabling the identification of potential strengths, limitations, and biases. For instance, XAI can compare medically known reference points with those used by AI during generation, ensuring that the model has learnt relevant features integral to the medical domain. Additionally, as AI models are trained through machine learning, XAI can evaluate the datasets used to train AI models by applying activation maximisation techniques like investigating the features a model’s layer focuses on [59]. However, advancing XAI still requires significant progress, including the development of more sophisticated techniques and the establishment of industry standards to ensure consistency and comparability across applications [60].

Another limitation is the high rate of false positives, as noted by Cui et al., 2022 [35]. Models optimised for sensitivity frequently flag benign or irrelevant findings as potential lesions, which can lead to unnecessary follow-up tests, increased healthcare costs, and added stress for patients. Therefore, further refinement is essential before these AI models can be broadly implemented in clinical settings.

### 4.5. Review Limitations

This systematic review has several limitations. First, the heterogeneity in study designs, AI model architectures, dataset compositions, and outcome reporting limited our ability to conduct a formal meta-analysis. Instead, we used descriptive summaries to synthesise findings across diverse studies, which may limit statistical precision. Second, although we employed a comprehensive multi-database search strategy, we restricted inclusion to English language articles, potentially omitting relevant non-English studies. Third, our exclusion of studies using only open-source datasets—while justified to reduce bias—may have excluded some well-performing algorithms that lacked access to local data. Fourth, the limited number of included studies (n = 14) reflects the strict quality and inclusion criteria but may reduce the breadth of generalisability.

Finally, despite ending our search in December 2022, this review was submitted in early 2025 and thus does not reflect the most recent developments in the field. However, as discussed, the slightly over two-year lag is consistent with translational timelines in medical AI and does not invalidate the relevance of the included studies. Future updates of this review will be needed as more validated, externally tested AI models emerge in the literature.

### 4.6. Future Directions

The WHO supports early detection programmes and encourages countries to implement screening measures for high-risk populations to detect lung cancer at earlier stages when treatment options are more effective [2]. While LDCT is the recommended imaging modality, its implementation faces challenges due to infrastructure, workforce shortages, and financial constraints in many low- and middle-income countries (LMICs) [61]. In this context, AI models that can support or partially automate nodule detection and malignancy classification offer a potential solution to scale up screening coverage without proportionally increasing radiologists’ workload. However, the WHO has also cautioned against the premature deployment of AI in healthcare without proper clinical validation, impact assessments, and regulatory oversight. Therefore, while AI-enhanced screening aligns with the WHO’s vision for equitable digital health, its integration into public health strategies must be guided by robust evidence and ethical safeguards.

Additionally, future initiatives to improve LCS should also emphasise leveraging technology and AI to enhance the interpretation of LDCT scans, the accuracy of lung nodule detection, and the classification of lung nodules [7]. Interestingly, smoking cessation efforts tend to be more successful when a nodule is detected on a CT scan, regardless of whether the nodule is benign or malignant [62]. This shows that LCS presents a unique opportunity for intervention, as the majority of those enrolled in LCS programmes are current smokers [7]. Therefore, in the future of integrating LDCT screenings with smoking cessation programmes, there could be an unintended yet highly valuable benefit of encouraging smokers to quit. This could potentially lead to an increase in early-stage lung cancer detection, improved prognosis, and decreased overall mortality of lung cancer patients in the long run [63].

## 5. Conclusions

This systematic review affirms the growing capabilities of AI in accurately classifying and detecting pulmonary nodules on CT thorax. While existing AI models have demonstrated considerable diagnostic potential, especially with the idea of enhancing early lung cancer detection, their clinical applicability remains constrained by challenges in their robustness and seamless integration into healthcare systems. Key limitations include the need for large, diverse training datasets, improved interpretability, and consistent validation of AI findings. Being able to address these gaps through the proper standardisation of the methodology and interdisciplinary collaboration can help to ensure the ethical and effective integration of AI systems into imaging practice, such as XAI. Therefore, continuous refinement and testing are crucial to transform these technologies from research settings to widespread clinical use.

## Figures and Tables

**Table 1:** Baseline characteristics.

Authors, Year	Study Type	CT Parameters	AI Model	Number of Nodules	Detection ^a^- or Classification ^b^-Focused
Gao et al., 2022 [32]	Retrospective	Not included	ResNet50 network	330	Detection
Katase et al., 2022 [33]	Retrospective	2 mm slice thickness	Faster R-CNN	115	Detection
Abadia et al., 2022 [34]	Retrospective	1 mm slice thickness	AI-RAD Companion Chest CT	441	Detection
Cui et al., 2022 [35]	Retrospective	Not included	2× CNN with VCG-net architecture	262	Detection
Hsu et al., 2021 [36]	Retrospective	512 × 512 matrix, 2.5 mm slice thickness	ClearReadCT	340	Detection
Guo et al., 2020 [37]	Retrospective	1.5 mm (thin subset), 5 mm (thick subset)	DeepLN DNN	766	Both
Kozuka et al., 2020 [38]	Retrospective	1 mm slice thickness	CAD-InfeRead CT Lung, Faster R-CNN	743	Detection
Qiu et al., 2022 [39]	Retrospective	512 × 512 matrix, 1–1.5 mm slice thickness	DenseNet	254	Classification
Diao et al., 2022 [40]	Retrospective	Not included	Unnamed in-house model	431	Classification
Du et al., 2022 [41]	Retrospective	512 × 512 matrix, 1 mm slice thickness	Unnamed in-house model	194	Classification
Marappan et al., 2022 [42]	Retrospective	Not included	2D/3D Dense-Net, Softmax	195	Classification
Lv et al., 2021 [43]	Retrospective	<2.5 mm slice thickness	FGP-NET	100	Classification
Heuvelmans et al., 2021 [44]	Retrospective	Not included	LCP-CNN	2106	Classification
Pang et al., 2020 [45]	Retrospective	Not included	DenseNet + AdaBoost	3940	Classification

^a^ Detection refers to the identification and localization of pulmonary nodules on CT images, i.e., whether a lesion is present or not; ^b^ classification refers to the assessment of the likelihood that a detected nodule is malignant or benign based on its imaging characteristics.

**Table 2:** Consolidated results for articles reviewed in the “Detection” subgroup.

Articles for Detection						
Author	AI Sen	Rg Sen	AI Spe	Rg Spe	AI Acc	Rg AUC
Gao et al., 2022 [32]	95.5	-	-	-	-	-
Katase et al., 2022 [33]	98.1	75	-	-	-	-
Abadia et al., 2022 [34]	96.1	-	77.5	-	90.9 ^c^	-
Cui et al., 2022 [35]	90.1 (86.4–93.7)	76 (70.7–81.2)	-	-	-	-
Hsu et al., 2021 [36]	86	74 (72–77)	87	87 (85–89)	85.71	81
Guo et al., 2020 [37]	96.5 (thin), 89.6 (thick) ^d^	-	-	-	99.02	-
Kozuka et al., 2020 [38]	95.5 (89.9–98.5)	68 (61.4–74.1)	83.3 (35.9–99.6)	91.7 (61.5–99.8)	-	-

^c^ Value was calculated manually from true/false positive/negative values provided; ^d^ values were separated into subsets for scans of thin and thick sections; AI: artificial intelligence; Acc: accuracy; AUC: area under curve; Rg: radiologist; Sen: sensitivity; Spe: specificity.

**Table 3:** Summary statistics for the “Detection” subgroup.

	Range	Mean ^e^	Standard Deviation ^e^
AI Sensitivity	86.0–98.1	94	3.99
Radiologist Sensitivity	68–76	73.3	3.11
AI Specificity	77.5–87	82.6	3.91
Radiologist Specificity	87–91.7	89.4	2.35
AI Accuracy	85.7–99.0	91.9	5.48

^e^ All values were rounded off to 3 significant figures, where applicable; AI: artificial intelligence.

**Table 4:** Consolidated results for articles reviewed under the “Classification” subgroup.

Articles for Classification								
Author	AI Model	AI Sen	Rg Sen	AI Spe	Rg Spe	AI Acc	Rg Acc	AI AUC
Qiu et al., 2022 [39]	DenseNet	60.58 (53.58–67.28)	76.27	84.78 (71.13–93.66)	61.67	64.96 (58.75–70.82)	73.31	77.6 (70.4–84.8)
Diao et al., 2022 [40]	Unnamed in-house model	-	-	-	-	-	-	-
Du et al., 2022 [41]	Unnamed in-house model	92.88	88.3	65.22	65.22	89.69	85.57	76.8
Marappan et al., 2022 [42]	2D/3D Dense-Net, Softmax	74.4	-	90	-	86.67	-	-
Lv et al., 2021 [43]	FGP-NET	93.3 (85.3–97.1)	86.7 (77.2–92.6)	64 (44.5–79.8)	84 (65.3–93.6)	-	-	92.7 (85.7–96.9)
Heuvelmans et al., 2021 [44]	LCP-CNN	-	-	-	-	-	-	94.5 (92.6–96.1)
Pang et al., 2020 [45]	DenseNet + AdaBoost	-	-	-	-	89.9	-	-
Guo et al., 2020 [37]	DeepLN DNN	-	-	95.93	-	92.46	-	-

AI: artificial intelligence; Acc: accuracy; AUC: area under curve; Rg: radiologist; Sen: sensitivity; Spe: specificity.

**Table 5:** Summary statistics for the “Classification” subgroup.

	Range	Mean ^f^	Standard Deviation ^f^
AI Sensitivity	60.6–93.3	80.3	13.7
Radiologist Sensitivity	76.3–86.7	83.8	5.32
AI Specificity	65.2–95.9	80	13
Radiologist Specificity	61.7–84	70.3	9.79
AI Accuracy	65.0–92.5	84.8	10
Radiologist Accuracy	73.3–85.6	79.5	6.15
AI AUC	76.8–94.5	85.4	8.23

^f^ All values were rounded off to 3 significant figures, where applicable; AI: artificial intelligence; AUC: area under curve.

## Data Availability

Data sharing is not applicable. No new data were created or analyzed in this study.

## Abbreviations

The following abbreviations are used in this manuscript:

LDCT	Low-Dose Computed Tomography
LCS	Lung Cancer Screening
CXR	Chest X-Ray
AUC	Area Under Curve
XAI	Explainable Artificial Intelligence
AI	Artificial Intelligence
CT	Computed Tomography
